# Is it realistic to use microbial photosynthesis to produce electricity directly?

**DOI:** 10.1371/journal.pbio.3001970

**Published:** 2023-03-02

**Authors:** Christopher J. Howe, Paolo Bombelli

**Affiliations:** Department of Biochemistry, University of Cambridge, Cambridge, United Kingdom

## Abstract

It is now possible to generate small amounts of electrical power directly from photosynthetic microorganisms. Can this electricity be used outside of the lab, and what are the hurdles to be overcome?

## Teaser

It is possible to generate small amounts of electrical power directly from photosynthetic microorganisms—arguably the greenest of green energy. But will it have useful applications, and what are the hurdles if so?

There has long been interest in using microorganisms to generate electricity directly, in biologically driven electrochemical systems. The first such systems were operated with heterotrophic microorganisms and are known as microbial fuel cells. They rely on some of the electrons generated during metabolism being exported from the cell and collected by an anode. Microbial fuel cells offer the attractive possibility of simultaneously breaking down waste material and producing electricity, and have been used, for example, to produce power to illuminate lavatories from urine harvested there [1]. More recently, systems have been described that use photosynthetic microorganisms, rather than heterotrophs, to generate electricity [2–5]. How do they work and will they ever be useful?

Typical devices [2–4], referred to as “biophotovoltaic devices” or “BPVs” use oxygenic photosynthetic microorganisms (usually cyanobacteria, but eukaryotic algae can also be used). These organisms utilise solar energy to oxidise water, producing electrons that are usually used for carbon dioxide fixation within the cell, and oxygen as a waste product. However, some of the electrons leave the cell (“exoelectrogenesis”). The route the electrons take and the reason(s) why some electrons leave the cell are not clear. Exoelectrogenesis might help in metal mobilisation or in dealing with the effects of absorbing excess light energy. Nevertheless, the electrons can be collected by an anode, pass round an external circuit and recombine at a catalytic cathode with oxygen and protons to form water. During their passage round the external circuit, the electrons do useful work. Unlike conventional photovoltaic cells, BPVs also produce power in the dark (probably by metabolism of stored photosynthesis products), and unlike batteries, they don’t inevitably run down as they are powered by sunlight rather than the depletable redox couples of electrodes in batteries.

That is all very well in the lab, but will BPVs powered by photosynthetic microorganisms ever have real-world applications, and how soon? Laboratory studies have shown maximum power outputs in the region of 0.5 to 0.8 watts per square meter [5,6] and estimates suggest that they would in principle be able to produce up to a few watts per square meter. This is less than from a conventional photovoltaic installation, although by only a few-fold at most [3]. Experimental BPVs used in the lab so far are small, but have been able to power items including LEDs or small environmental sensors, and we recently reported that a BPV approximately the size of an AA battery could drive an ARM Cortex M0+ microprocessor stably for over 6 months [7]. This is a widely used low-power microprocessor and the power demand in this example (to sum repeatedly a series of integers and confirm the total calculated) was less than a microwatt.

**Fig 1:**
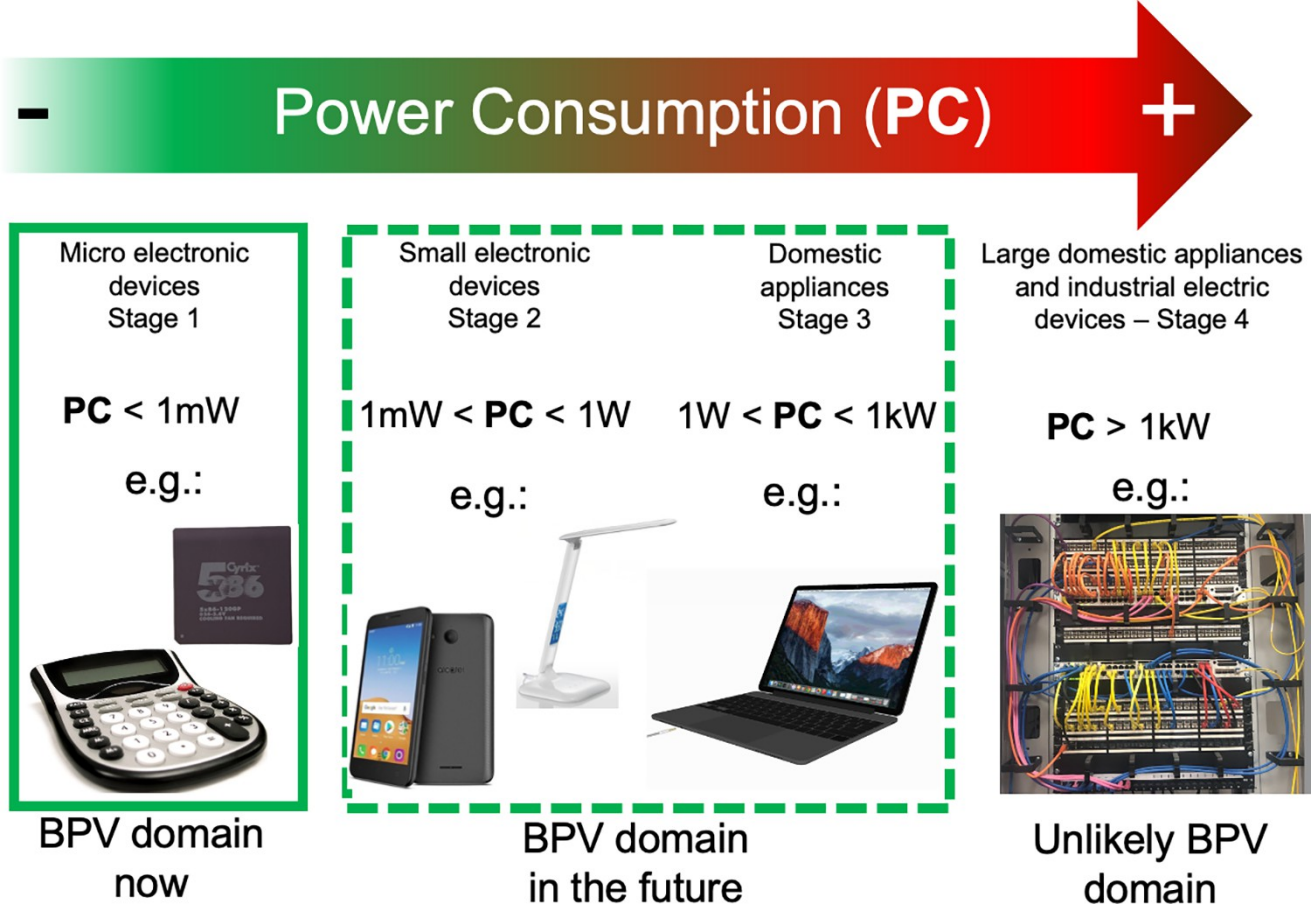
Present and future domains for applications of BPV. The figure suggests a range of applications, from those which could be powered by BPV at present (Stage 1, left), through to applications for which powering by BPV, even in the future, is unlikely (Stage 4, right).

With the power densities presently achieved, BPVs could in principle be used now to run small devices with low power requirements (up to a mW or so) (Fig 1, Stage 1). They would be particularly useful to run things that cannot readily be run from a local power supply and might otherwise rely on batteries or solar cells. Examples might include small electronic gadgets in domestic environments, such as digital clocks, thermometers, and microprocessors in the Internet of Things. Applications outside domestic environments might include environmental sensors in remote locations, for example, in monitoring water quality [8]. BPVs would have an advantage over solar cells if power were needed in the dark as well as the light. Scaling up of devices to provide milliwatts or a watt or two also seems feasible in principle, although scale-up may take a few years. This would open up applications such as running mobile phone chargers or low-level lighting (Stage 2). These might be particularly attractive in rural areas of low- or middle-income countries (LMICs) or in disaster relief, where small amounts of power, and the ability to charge a phone, could be particularly useful. It’s worth noting that in many LMICs, mobile phones are an increasingly important resource for healthcare delivery [9].

It might become feasible to run small domestic appliances such as laptops, especially in off-grid locations (Stage 3), but this will require improvements in the performance of BPVs. We think it is unlikely to be feasible to run larger domestic or industrial appliances with BPVs (Stage 4), whether the power is generated locally or supplied by a grid, because of the large surface area that would be needed for power generation. For the same reason, it is even less likely to be feasible to incorporate BPVs into transport vehicles to power them. The surface area required and the resulting weight would be too great.

How can we improve the performance of BPVs? We need to optimise power output while using sustainable materials. Highly structured anodes containing indium and tin are efficient [2], but use of these in large quantities may not be sustainable. The BPV used to drive the microprocessor [7] used an aluminium anode, and although the power density may be lower than with structured anodes, aluminium anodes can be made from waste, such as drinks cans. This makes them an attractive choice. (The rest of the BPV shell can also be made from waste material such as plastic bottles [10].) Using optimised microbial strains will also enhance output, though for many installations genetically modified organisms may be unacceptable. The best strains will probably be isolated from the locations where the devices are to be used. We also need to think about the cathode. Most BPVs at present use catalytic platinum. The amount is small (less than 1 mg cm^−2^ of cathode, which is equivalent to only a few cents or pence for the microprocessor BPV [7]) and it should be recyclable, but we should look for other materials—or use microorganisms as biocathodes.

What other hurdles are there? There is a tendency among researchers to judge BPVs against conventional photovoltaics purely in terms of their power generation when operating. However, comparisons are more complicated than that. One needs to assess the whole life cycle—building, decommissioning and recycling the components of the devices. The reliance of BPVs on self-replicating organisms (ideally isolated from the environment where they will be used) rather than energy-intensive photovoltaic materials is a particular attraction. Once we have a better idea of how BPVs will be built for use in the real world (and this will be informed by studies on the initial applications suggested above), we can carry out the life cycle assessments that will help us judge objectively the benefits of BPVs. It will also be essential to understand the needs of local communities where BPVs are to be installed, especially in LMICs, and to involve the communities closely. A third challenge will be to persuade stakeholders (including research funders) to switch to a novel technology like BPVs. In spite of all these challenges, we believe that successful applications of BPVs in the lab suggest they can contribute to energy provision in the near future, especially for situations requiring small amounts of power without a direct supply.
